# Reversal of Hyperglycemia and Suppression of Type 1 Diabetes in the NOD Mouse with Apoptotic DNA Immunotherapy™ (ADi™), ADi-100

**DOI:** 10.3390/biomedicines8030053

**Published:** 2020-03-04

**Authors:** David G. Alleva, Melika Rezaee, Linda Yip, Gang Ren, Jarrett Rosenberg, Waldo Concepcion, Alan Escher, Shahrokh Shabahang, Avnesh S. Thakor

**Affiliations:** 1 Aditx Therapeutics, Inc., 11161 Anderson Street, Suite 105-10014, Loma Linda, CA 92354, USA; dalleva@immucrine.com; 2 Interventional Regenerative Medicine and Imaging Laboratory, Department of Radiology, Stanford University School of Medicine, Palo Alto, CA 94304, USA; melrezaee@gmail.com (M.R.); tgren@stanford.edu (G.R.); Jarrett.Rosenberg@stanford.edu (J.R.); waldo1@stanford.edu (W.C.); 3 Division of Immunology and Rheumatology, Department of Medicine, Stanford University, Stanford, CA 94305, USA; lindayip@stanford.edu; 4 Department of Basic Sciences, School of Medicine, Loma Linda University, Loma Linda, CA 92354, USA; sshabahan@aditxt.com

**Keywords:** diabetes, immunotherapy, hyperglycemia, DNA immunotherapy, NOD mouse, type 1 diabetes, antigen-specific, DNA plasmids, ADi-100, monotherapy, apoptosis, BAX, T cells, sGAD55, pre-diabetic mouse

## Abstract

The antigen-specific apoptotic DNA immunotherapeutic, ADi-100, is designed to suppress type 1 diabetes and consists of two DNA plasmids encoding genetic sequences of the apoptosis-inducing molecule, BAX, and the secreted form of the autoantigen, glutamic acid decarboxylase 65, that is CpG hyper-methylated to avoid inflammatory signaling (msGAD55). Upon a four-day treatment with ADi-100 of young female non-obese diabetic (NOD) mice, the frequency of various tolerogenic dendritic cell populations increased in draining lymph nodes; these cells lost the capacity to stimulate glutamic acid decarboxylase (GAD)-specific CD4^+^ T lymphocytes and were associated with the previously demonstrated enhancement of GAD-specific regulatory T cells. The efficacy of two ADi-100 formulations containing different proportions of BAX and msGAD55, 1:4 (10/40 µg) and 1:2 (17/33 µg), was evaluated in mildly hyperglycemic pre-diabetic NOD female mice. Both formulations suppressed the incidence of diabetes by 80% in an antigen-specific manner, while all untreated mice developed diabetes. However, treatment of pre-diabetic mice with significantly higher hyperglycemia, denoting progressive disease, showed that ADi-100 1:2 strongly suppressed diabetes incidence by 80% whereas the ADi-100 1:4 was less effective (50%). As an antigen-specific monotherapy, ADi-100 is highly efficacious in reversing elevated hyperglycemia to prevent diabetes, in which increasing apoptosis-inducing BAX content is a promising immune tolerance feature.

## 1. Introduction

Type 1 diabetes mellitus (T1D) is an autoimmune disease in which insulin-producing β-cells within pancreatic islets are destroyed by an autoimmune attack coordinated by autoantigen-specific polyclonal T lymphocytes that have escaped control of immune tolerance [1,2]. The field of immunotherapeutics is addressing defective tolerance processes with antigen-specific immunotherapies (ASIs) that have vaccine-like qualities that avoid unwanted effects characteristic of broad-acting immunosuppressive therapeutics. A promising class of ASIs utilize the natural cell death process, apoptosis [3,4,5,6], which is a natural non-inflammatory tolerance-inducing pathway. Antigen-presenting cells (APCs), such as dendritic cells (DCs), become tolerogenic after engulfing apoptotic cells; this enables the presentation of processed apoptotic cell autoantigens (without co-stimulation) to regulatory T cells (Tregs) for stimulation or to autoreactive memory effector T cells (Teff) for inactivation [3,4,5,6]. Our lab has developed a unique and potent ASI, ADi-100, that consists of two DNA plasmids, one expressing the intracellular apoptosis-inducing signaling molecule, BAX, and the other expressing the islet autoantigen, secreted glutamic acid decarboxylase 65 (sGAD55) [3,7,8]. We have previously shown that the efficacy of ADi-100 in the non-obese diabetic (NOD) mouse model of T1D is significantly increased if the sGAD55 plasmid is hyper-methylated [8], which is intended to reduce inflammation caused by unmethylated CpG motifs that are ligands for the Toll-like receptor 9 expressed on some APCs. ADi-100 treatment also increases sGAD-specific Treg levels in draining lymph nodes of NOD mice along with total CD11c^+^ DCs [7,8,9]; although it is not known whether these DCs have a tolerogenic phenotype. In this study, ADi-100 treatment increased tolerogenic DCs (tol-DCs), and increasing the apoptosis-inducing BAX content enhanced the efficacy in reversing hyperglycemia when administered to NOD mice during late hyperglycemia, a pre-diabetes stage that has relevance to the corresponding clinical diagnosis stage in human T1D.

## 2. Materials and Methods

### 2.1. ADi-100: Plasmid DNA Construct

The two DNA plasmids that comprise the ADi-100 formulation previously described [8] are pND2-BAX containing a *bax* cDNA sequence under transcriptional control by the CMV promoter and pSG5-GAD55 containing a cDNA construct encoding a secreted form of human GAD65 (sGAD55) under transcriptional control of the SV-40 promoter in the pSG5 vector (Stratagene, San Diego, CA, USA). The pSG5-GAD plasmid was hyper-methylated at CpG motifs (msGAD55) in *Escherichia coli* strain, ER1821, via the activity of SssI methylase (New England BioLabs, Ipswich, MA, USA). Plasmid DNA was dissolved in sterile saline immediately prior to intradermal (i.d.) injection. Consistent with our previously published results [9], all plasmids containing the *bax* sequence insert showed significant and substantial degrees of apoptosis of human HeLa cells (using 1 ug/mL DNA in cultures; data not shown), confirming the activity of the BAX-induced apoptosis tolerance delivery system of ADi-100.

### 2.2. Animals

Eight-week-old female NOD mice were purchased from Taconic Farms (NOD/MrkTac; Germantown, NY, USA) for studies at Loma Linda University (Loma Linda, CA, USA) [8] and from The Jackson Laboratory (NOD/ShiLtJ; Sacramento, CA, USA) for studies at Stanford University (Palo Alto, CA, USA). All animals were housed in vivariums under pathogen-free conditions at their respective locations and experimentation was approved by the respective Institutional Animal Care and Use Committees.

### 2.3. Dendritic Cell Isolation and Characterization

Eight-week-old female NOD mice (Taconic Farms) received 2 i.d. injections of 50 µg plasmid DNA alone (vector) or ADi-100 1:4 (BAX 10 µg + msGAD 40 µg) in the abdominal flank region 7 days apart, and leukocytes were isolated from draining inguinal lymph nodes 4 days after the second injection, at which time single-cell suspensions were prepared for analysis of various DC phenotypic populations via flow cytometry. These freshly isolated cells (10^6^) were incubated with one or more of the following conjugated antibodies (1 µg; see below) for 30 min on ice and evaluated using a FACSCalibur (BD Biosciences, Franklin Lakes, NJ, USA) as previously described [7]; rat anti-mouse CD317/PDCA-1, clone 129C1, PE-conjugated (BioLegend, San Diego, CA, USA); hamster anti-mouse CD11c, clone N418, FITC-conjugated (BioLegend, San Diego, CA, USA); rat anti-mouse MHC Class II, clone M5/114.15.2, APC-conjugated (R&D Systems, Minneapolis, MN, USA); rat anti-mouse CD8a, clone 53–6.7, PE-conjugated (BioLegend, San Diego, CA, USA); rat anti-mouse Integrin αM/CD11b, Clone M1/70, Alexa Fluor 647 conjugated (R&D Systems, Minneapolis, MN, USA); rat anti-mouse CD103, Clone M290, PE-conjugated (BD Biosciences, Franklin Lakes, NJ, USA); rat anti-mouse CD207, Clone 4C7, PE-conjugated (BioLegend, San Diego, CA, USA).

### 2.4. GAD-Specific T Lymphocyte Proliferation

To evaluate the ADi-100-induced tolerogenic properties of DCs, pooled splenocytes from eight ADi-100-vaccinated NOD mice (as described above) were used to isolate CD11c^+^ (cDC; CD11c^+^ CD8^+^ Integrin αvβ8^+^) and CD11c^−^ (plasmacytoid DCs, pDC; CD11c-/PDCA+) tol-DC populations using the CD11c positive and mPDCA-1 positive kits (Miltenyi, Auburn, CA, USA), respectively. GAD-stimulated CD4+ lymphocytes were generated by culturing 10^6^ lymph node cells from 8-week-old female NOD mice with GAD (20 µg/mL) in 1 mL of culture medium (Dulbecco’s modified Eagle’s medium with high glucose, DMEM; Sigma, St. Louis, MO, USA) supplemented with 10% heat-inactivated fetal bovine serum (FBS; HyClone, Logan, UT, USA), 2 mM L-glutamine, 1 mM sodium pyruvate, 0.11 mM sodium bicarbonate] for 3 days, after which CD4+ T cells were enriched as untouched cells using negative selection with anti-CD8, -CD11b, -CD16, -CD56, -CD19, and -CD36 mAbs (Miltenyi Biotec, Auburn, CA, USA), as previously described [7]. T cell purity assessed via flow cytometry was >95% (data not shown). GAD-stimulated CD4+ T cells were stained with 1.5 uM CFSE (Invitrogen, Carlsbad, CA, USA) prior to culture with DCs. DCs (5 × 10^4^) were cultured with CD4^+^ T cells (5 × 10^4^) and hrIL-2 (20 U/mL; PeproTech, Rocky Hill, NJ, USA) in the presence or absence of sGAD (20 µg/mL, generated at Loma Linda University by A.E.) in triplicate wells of 96-well plates. After 72 h of culture, anti-CD4-PE mAb and the green nucleic acid stain dead cell-indicator, SYTOX^®^, (Invitrogen, Carlsbad, CA, USA) were used to detect CFSE^+^CD4^+^SYTOX^−^ cell proliferation via flow cytometry per the manufacturer’s instructions. FlowJo 7.6.5 software (Becton, Dickinson, & Co., Ashland, OR, USA) was used to analyze proliferation data, and the percentage of divided CD4+ T cells represents the degree of proliferation. The percentage of divided cells in the absence of sGAD antigen was <1% (not shown).

### 2.5. Diabetes Studies in NOD Mice

Two NOD mouse diabetes studies were performed by two separate laboratories, respectively, to demonstrate the robustness of ADi-100 efficacy: The first study with mildly hyperglycemic female NOD mice was performed at Loma Linda University (Loma Linda, CA, USA) [8] and the second study with highly hyperglycemic female NOD mice was performed at Stanford University (Palo Alto, CA, USA). All animals were purchased at 8 weeks of age and blood glucose levels were monitored weekly with a glucometer (Bayer Contour Glucose Meter; Ascensia Diabetes Care, Parsippany, NJ, USA) as previously described [8]. Upon the first reading ≥140 mg/dL (fasting blood glucose, FBG, mildly hyperglycemic study) or upon at least two readings ≥180 mg/dL or upon the first occurrence ≥200 mg/dL (morning blood glucose, mBG, highly hyperglycemic study), the animals were randomly assigned to cohorts to receive the first weekly injection of ADi-100 (50 µg) or control vectors. Animals received 50 µL i.d. injections into the abdominal flank as previously described [8], and blood glucose levels were monitored weekly in which diabetes was diagnosed when blood glucose was ≥300 mg/dL on two occasions at least 7 days apart. In the mildly hyperglycemic study, each diabetic mouse was euthanized when FBG reached ≥600 mg/dL, and those that were diabetes-free were euthanized at 50 weeks of age. In the highly hyperglycemic study, all animals were euthanized at 5 weeks post treatment to obtain and compare tissue samples at the same time point. Because the mildly and highly hyperglycemic studies entailed blood glucose assessments as FBG and mBG, respectively, we determined the true mean and SEM difference to be 17.9 ± 10 mg/dL, with FBG being intuitively less than the respective mBG reading due to fasting (two mBG readings were assessed the day before and the day after the respective FBG reading, which was evaluated once per week for 7 weeks for each of the 4 non-diabetic mice; i.e., a total of 28 FBG readings and 56 mBG readings that yielded 56 Δ values was used in deriving the mean difference).

### 2.6. Immunohistochemistry

Animals were euthanized at the end of the experiment and pancreata harvested, embedded in OCT compound (Tissue Tek, Torrance, CA, USA) or paraffin, and stained for insulin using a rat anti-insulin primary antibody ((1:100, #MAB1417, R & D systems, Minneapolis, MN, USA) and a donkey anti-rat IgG secondary antibody conjugated to Alexa488 (1:500, Invitrogen, Carlsbad, CA, USA) as previously described [10].

### 2.7. Statistical Analysis

Kaplan–Meier estimates of the disease-free survival curves were plotted and differences among groups were tested by log rank test. Comparisons of continuous variables between groups were performed with Wilcoxon tests; comparisons of categorical variables were performed with Fisher’s exact test. All data were analyzed with Stata Release 15.2 (StataCorp LP, College Station, TX, USA). A significance level of 0.05 was used. The two-tailed *t* test (Prism, GraphPad Software, Inc, San Diego, CA, USA) was used to compare means.

## 3. Results

### 3.1. Tol-DC Subset Analysis in Draining Lymph Nodes after ADi-100 Treatment

**Figure 1:**
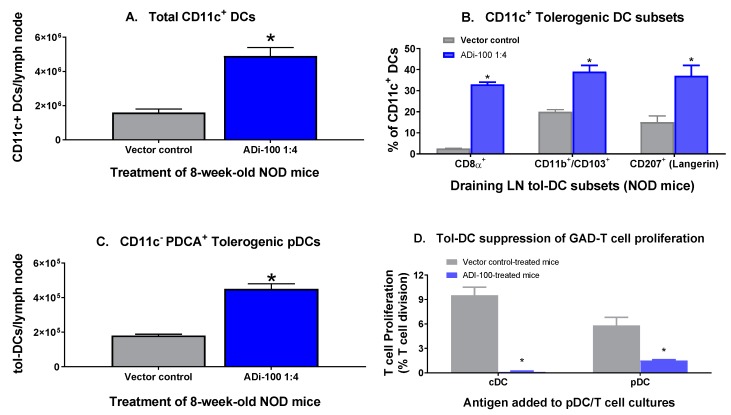
ADi-100-induced tol-DC subsets in draining lymph nodes of NOD mice. Groups of 8-week-old female NOD mice were vaccinated with vector plasmid DNA alone (control) or ADi-100 1:4 (BAX 10 µg + msGAD55 40 µg). After 4 days of vaccination, leukocytes were isolated from draining lymph nodes (inguinal) and DC populations were analyzed via flow cytometry. The following are phenotypic definitions of DC populations: total classical DC population, MHC Class II^+^/CD11c^+^ (**A**), tol-DC lymphoid tissue-resident populations, MHC Class II^+^/CD11c^+^/CD8α^+^ (**B**) and MHC Class II (IAg^7^)^+^/CD11c^−^/PDCA^+^ (plasmacytoid DC); (**C**), and tissue-migratory/Non-lymphoid tissue tol-DC populations, MHC Class II^+^/CD11c^+^/CD207^+^ (**B**) and MHC Class II^+^/CD11c^+^/CD11b^+^/CD103^+^ (**B**). * *p* < 0.001 compared to vector control cohort (two-tailed *t* test). CD11c^+^ (cDC; CD11c^+^ CD8^+^ Integrin αvβ8^+^) and CD11c^−^ (plasmacytoid DCs, pDC; CD11c-/ PDCA^+^) tol-DC populations prepared from splenocytes of vector control- or ADi-100 1:4-treated NOD mice were cultured with GAD-stimulated (3-day) CD4+ T lymphocytes from untreated NOD mice and rhIL-2 for 72 h and proliferation was assessed via CSFE staining and flow cytometry (**D**). Cell division was analyzed using FlowJo software and proliferation is reported as the percentage of dividing cells per total CD4^+^ T cells.

The BAX component of ADi-100 was designed to induce tol-DC migration to draining lymph nodes that subsequently present antigen to stimulate GAD-specific Treg cell numbers and function. Indeed, we have previously shown that delivery of a plasmid containing BAX and sGAD55 induced functional GAD-specific Treg cells in draining lymph nodes in NOD mice [7], in addition to increasing the number of total CD11c^+^ DCs in draining lymph nodes and spleen [9]. Here, we further defined the “tolerogenic” phenotypes of such DCs (different tol-DC phenotypes reviewed in [11,12]) by evaluating tol-DC populations four days after the second of two weekly injections of ADi-100 1:4 via flow cytometric analysis of draining inguinal lymph nodes (Figure 1). While it was confirmed that the total CD11c^+^/MHC class II^+^ DC population per lymph node increased by 3-fold (Figure 1A), strikingly, the CD8α^+^ tol-DC proportion of the total CD11c^+^ population increased by 13-fold, while the CD11b^+^/CD103^+^ and CD207^+^ tol-DC proportions of the CD11c^+^ population increased by 2- and 2.5-fold, respectively (Figure 1B). Furthermore, the number of tolerogenic plasmacytoid DCs (pDC; CD11c^−^/ PDCA^+^) per lymph node increased by 2.5-fold (Figure 1C). These results demonstrate that ADi-100 significantly and substantially increased tol-DC migration to the inguinal draining lymph nodes of the abdominal flank injection site. These phenotypically defined DC populations were further evaluated for tolerogenic activity on GAD-specific CD4+ T lymphocyte proliferation. Both CD11c^+^ (cDC; CD11c^+^ CD8^+^ Integrin αvβ8^+^) and CD11c^−^ (plasmacytoid DCs, pDC; CD11c-/ PDCA^+^) tol-DC populations prepared from splenocytes of vector control- or ADi-100-treated NOD mice lost their ability to support proliferation of GAD-stimulated CD4^+^ T lymphocytes (Figure 1D), consistent with a tolerogenic phenotype.

### 3.2. Efficacy of ADi-100 Containing Increased BAX Plasmid Content to Reverse Hyperglycemia in Mildly Hyperglycemic NOD Mice

**Figure 2:**
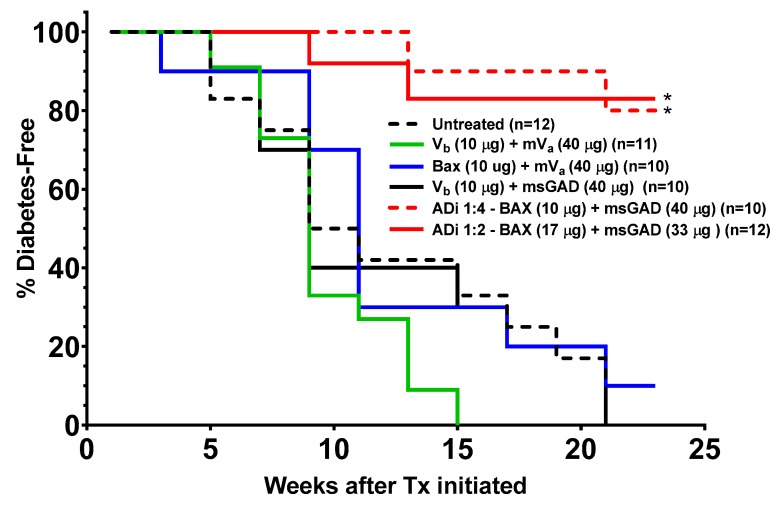
Two ADi-100 formulations containing different BAX and msGAD55 content suppressed the incidence of diabetes in NOD mice when treating mild hyperglycemia (≥140 mg/dL). Groups of 8-week-old female NOD mice were monitored weekly for fasting blood glucose (FBG) levels, and on the first day that FBG was ≥140 mg/dL (day 0; mild hyperglycemia), mice received an i.d. injection (50 µL) once per week for 8 weeks of formulations containing 50 µg total of different amounts of empty vectors (V_b_, BAX vector; mV_a_, methylated antigen vector) and those carrying BAX or msGAD55 [8]. Untreated mice did not receive any injection. The study was terminated once 100% of mice were diagnosed with diabetes in the untreated cohort (i.e., 2 FBG readings ≥300 mg/dL at least 7 days apart). The percentage of mice that remained free of diabetes in each cohort is presented. Note that raw FBG data per mouse used to calculate disease incidence for the first five cohorts (but not ADi-100 1:2) were obtained from data sets that appeared in our previous publication [8], but which were only presented as raw FBG data in a longitudinal format (mouse age); i.e., here, the data are represented in the form of “diabetes incidence” that includes the additional ADi-100 1:2 data that were not included in the previous publication. * *p* < 0.001 compared to untreated cohort.

Since BAX-induced apoptosis enhances immune tolerance, we evaluated whether increasing BAX plasmid content of our ADi-100 formulation could enhance the efficacy in reversing hyperglycemia in mildly hyperglycemic female NOD mice. Two ADi-100 formulations containing BAX content at a lower amount (10 µg BAX plasmid and 40 µg msGAD55 plasmid; i.e., 1:4 ratio of BAX:msGAD55) or higher amount (17 µg BAX + 33 µg msGAD55, 1:2 ratio, 50 µg total) were administered when FBG >140 mg/dL. While the untreated and the empty vector treated cohorts reached 100% diabetes incidence (except the mVa + BAX that reached 90%), there was a significantly lower incidence of diabetes in the ADi-100 1:2- and 1:4-treated cohorts of only 20% at 15 and 23 weeks, respectively (Figure 2). Note that none of the ADi-100-treated mice developed diabetes during the first eight weeks during ADi-100 administration (Figure 2). As there were no differences in efficacy between the ADi-100 1:4 and 1:2 formulations administered at a relatively early disease stage of mild hyperglycemia with a mean ± SEM FBG of 173 ± 4 mg/dL at day 0 of treatment, we further evaluated possible differences in efficacy by treating mice later in the disease process at significantly higher glycemic levels.

### 3.3. Increased BAX Plasmid Content in the ADi-100 1:2 Formulation Resulted in Greater Efficacy in a Later Stage of Autoimmune Diabetes (i.e., Highly Hyperglycemia)

A challenge in treating NOD mice to reverse hyperglycemia and suppress diabetes onset is to ensure that only mice likely to develop diabetes are treated, and that the timing of treatment is within the “pre-symptomatic” hyperglycemic stage just prior to disease onset when the extent of β-cell loss still permits reversal of hyperglycemia. We derived a true hyperglycemic threshold as 4 × SD above the mean of normal mBG levels of our aged diabetes-free female mice (mean ± SD, 113 ± 17 mg/dL; *n* = 685 daily mBG readings from five naturally diabetes-free mice; note that 20% of our colony remain diabetes-free), which was 180 mg/dL, above which a non-diabetic mouse is highly unlikely to have a mBG reading (*p* = 0.00003). Indeed, Mathews et al. [13] recently recommended that mBG values should be used instead of FBG to avoid any untoward influence of fasting on the course of disease progression. To determine ADi-100 efficacy when administered relatively late in the disease process during high hyperglycemia, each NOD mouse received the first ADi-100 dose when mBG was ≥ 180 mg/dL and weekly doses thereafter for a total of five injections. The mean ± SEM mBG on day 0 for all 31 mice was 244 ± 12 mg/dL, which was significantly greater than the FBG mean ± SEM of 173 ± 4 mg/dL of the mild hyperglycemic study (*p* < 0.001). Note that the inherent difference between FBG and mBG of 18 ± 10 mg/dL does not account for the large differential of these day 0 mean values (see Section 2.5).

**Figure 3:**
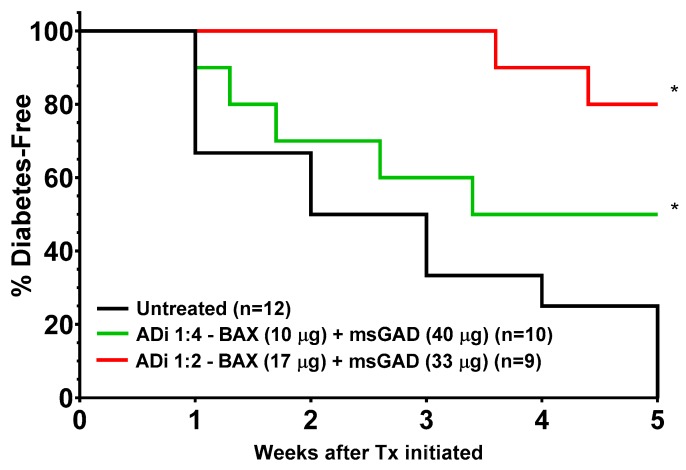
Increased efficacy of ADi-100 containing greater BAX plasmid content when administered to highly hyperglycemic NOD mice. Groups of female NOD mice were monitored weekly for morning blood glucose (mBG) levels, in which each mouse received the first ADi-100 dose (day 0) of an i.d. injection of either of two ADi-100 formulations, 1:4 or 1:2, when mBG was ≥180 mg/dL on at least two occasions or upon the first occurrence of mBG ≥200 mg/dL. On day 0, the mean ± SEM mBG of all 31 mice was 244 ± 12 mg/dL. Mice received weekly ADi-100 injections thereafter for a total of five injections. Daily mBG monitoring continued and mice were diagnosed with diabetes when ≥ 300 mg/dL on two occasions at least seven days apart. The percentage of mice that remained free of diabetes in each cohort are presented. * *p* < 0.035 for ADi-100 1:4 and *p* < 0.001 for ADi-100 1:2 compared to untreated cohort.

While the untreated cohort progressively developed diabetes showing an incidence of 100% by five weeks (i.e., day 35, study termination), the ADi-100 1:4 treated cohort showed an ultimate 50% suppression of disease incidence from day 17 to the end of the five-week study (Figure 3; vs. untreated, *p* = 0.035). Importantly, the ADi-100 1:2 treated cohort showed an 80% suppression of disease incidence from day 31 to the end of the study which was highly significant relative to the untreated group (*p* = 0.001), a statistical significance much greater than that of the ADi-100 1:4 treated group (*p* = 0.035). The probability between the ADi-100 1:2 and 1:4 groups was *p* = 0.17 due to an insufficient number of mice that “converted to diabetes”. It is apparent that the “highly hyperglycemic” acceptance criteria of ≥180 mg/dL led to the initiation of treatment substantially later in the disease process relative to the mildly hyperglycemic study because the time to 100% diabetes incidence in the untreated control groups was substantially faster in the highly hyperglycemic study; 5 vs. 23 weeks, respectively (Figure 2 and Figure 3).

**Figure 4:**
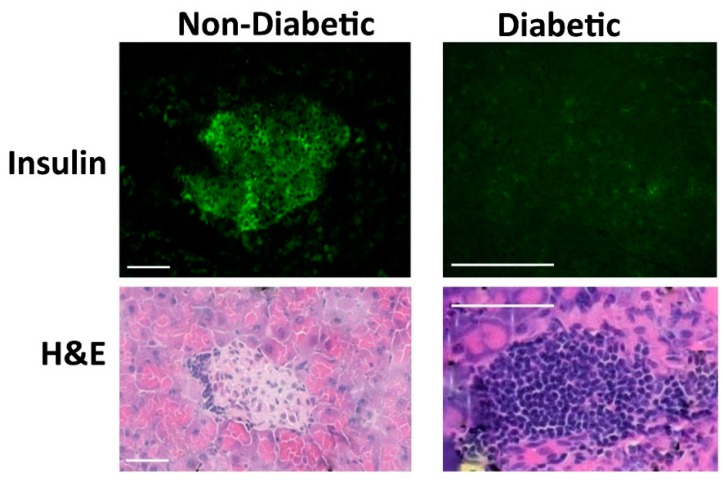
Immunohistochemical analysis of insulin (upper panels) and hematoxylin and eosin (H&E; insulitis) (lower panels) staining in pancreatic islet samples from representative untreated NOD mice that were non-diabetic (left panels) or diabetic (right panels). Scale bars = 50 μm.

Additional differences exist between the two ADi-100 formulations: (1) Although efficacy in the ADi-100 1:4 group appeared to show a bias of higher mBG day 0 values in the five non-responder mice, this theme did not appear to be the case with the ADi-100 1:2 formulation in which mouse #5 was protected from developing diabetes while having an exceptionally high mBG level of 286 mg/dL on day 0 (Table 1); (2) ADi-100 1:2 appeared to substantially extend the time from day 0 to T1D diagnosis relative to that of ADi-100 1:4 (mean of 4 days for the 1:4 cohort vs. 18 and 29 days for mice #1 and #2 in the 1:2 cohort; Table 1); (3) mBG levels of all five ADi-100 1:4 diabetic non-responders were ≥ 600 mg/dL, whereas those of the two from ADi-100 1:2 were controlled below this level at the end of the study (Table 1); and (4) pancreatic islet insulin expression analysis showed that ADi-100 1:2 responders (i.e., non-diabetic mice at day 35) were positive for insulin, whereas all three of the available samples from ADi-100 1:4 responders were negative (Table 1; see examples of positive and negative insulin staining in Figure 4). Interestingly, these insulin-negative samples of the three ADi-100 1:4 responders correlated with terminal mBG levels in the hyperglycemic range (≥180 mg/dL), whereas those of the ADi-100 1:2 responders were below this threshold, demonstrating that only ADi-100 1:2, but not ADi-100 1:4, reversed hyperglycemia (Table 1). Thus, this correlation of insulin staining with glycemic levels suggests that responder mice in the less potent ADi-100 1:4 formulation group may ultimately have developed diabetes if followed beyond day 35. Samples from the untreated diabetic control mice and those from all ADi-100 non-responder (diabetic) mice were negative for insulin staining at study termination (Table 1; some low signals from insulin staining were observed in 2 of 10 untreated diabetic mice at study termination; not shown).

## 4. Discussion

Collectively, our results demonstrate that ADi-100 induced tol-DC subset migration to draining lymph nodes and that it was strongly efficacious in reversing hyperglycemia and preventing the onset of diabetes in two independent studies; a mechanism that was antigen-specific and relied on the apoptosis-inducing factor, BAX, because neither plasmid alone was efficacious. Importantly, the robust efficacy of ADi-100 is evident in the reproducible results of experiments conducted at two different institutions, a concept that has been raised by the T1D research community [14]. Enhanced efficacy could be achieved by increasing the BAX content in the ADi-100 1:2 formulation while proportionally decreasing msGAD55 content to maintain a total dose of 50 µg for comparison with the ADi-100 1:4 formulation. Therefore, it appears that the autoantigen level can be spared for the benefit of increased apoptosis. Furthermore, the msGAD55 plasmid was hyper-methylated at CpG motifs to avoid inducing inflammatory signaling, but the BAX plasmid was not hyper-methylated (i.e., hypo-methylated) to ensure that CMV promoter activity was not compromised [8]. While it may appear counterintuitive that increasing such hypo-methylated plasmid content led to enhanced efficacy, it has been demonstrated that a relatively small amount of unmethylated CpG oligonucleotide added to a tolerant ASI can increase expression of the anti-inflammatory cytokine, IL-10, to promote tol-DC and Treg cell development and immune tolerance [15]. Moreover, the hyper-methylation used in developing ADi-100 is analogous to the single-plasmid ASI (expressing proinsulin II) containing recombinantly modified CpG to CpC motifs to avoid inducing inflammation [16], which reversed hyperglycemic NOD mice in addition to showing promising efficacy in T1D clinical trials [17].

It is well established that ASIs containing different tolerance delivery systems (TDSs) and autoantigens prevent diabetes when administered to young pre-hyperglycemic NOD mice, which is similar to Stage 1 in human T1D (i.e., autoantibody positive titers with no signs of dysglycemia; reviewed in [18]). However, there are very few published studies demonstrating that such ASIs (as monotherapies) can “reverse hyperglycemia” (i.e., Stage 2) in NOD mice [19]. This is in contrast to several non-specific immunomodulatory agents, such as anti-CD3 mAb, that have successfully reversed hyperglycemia in NOD mice, either alone or in combination with an ASI [13,19,20,21] and have recently been effective at delaying insulin production loss in pre-diabetic (i.e., dysglycemia, Stage 2) subjects [22]. However, unlike ASIs, these non-specific therapies may not induce durable tolerance and thus would require long-term dosing with associated safety concerns. Indeed, DNA-based ASIs that contain proinsulin II [16] or secreted GAD, such as our ADi-100 [7,8], have shown success in reversing hyperglycemia in NOD mice when used as monotherapies. A bivalent IgG Fc-MHC/GAD65 fusion protein, DEF-GAD, has also demonstrated such efficacy [23]. The striking effectiveness of these ASI monotherapies to reverse hyperglycemia may be due to prolonged antigen presence in vivo combined with unique features of each TDS.

Female NOD mice spontaneously developed diabetic hyperglycemia with an incidence of <100%, depending on the colony and laboratory; i.e., usually 70% to 90% incidence [24]. Such unpredictability can be statistically accounted for in “disease prevention” studies with young non-diabetic mice by increasing the number per cohort. However, fewer mice can be used in “hyperglycemia reversal” studies if mice are selected based on the likelihood of developing diabetes. In our study, we empirically derived a hyperglycemic threshold of 180 mg/dL mBG that predictably led to the development of diabetes, which was the upper limit of the true normal mBG range derived from female mice that never developed disease. Indeed, this threshold model was confirmed with the 100% incidence of diabetes in the untreated control group of 12 mice. Note that our accurate prediction of diabetes development in female NOD mice using this threshold is consistent with others who derived a normal mBG range < 170 mg/mL [16] or < 175 mg/dL [13] and used a diabetes diagnosis of two consecutive values ≥ 300 mg/dL or ≥ 400 mg/dL, respectively (almost all diabetic mice in our study were terminated at mBG ≥ 500 mg/dL). While it is difficult to translate these glycemic stages of NOD mice to those of human T1D, it is clear that ADi-100 could target treatment during clinically detectable dysglycemia (i.e., Stage 2, including hyperglycemia [25]) prior to overt clinical diabetes (Stage 3).

Several prevention or intervention clinical trials with ASI monotherapies have generally shown disappointing outcomes of preserving insulin production (i.e., stimulated C peptide) and improving glycemic measures (HbA1c and insulin usage) [26]. Most of these ASIs consisted of only autoantigens delivered via oral or mucosal (intranasal) routes which could be considered weak TDSs, or of other weak or irrelevant TDSs such as Alum (e.g., GAD-Alum; Diamyd Therapeutics; [27]) or incomplete Freund’s adjuvant (IFA) [28,29]. Alum may not be the most effective TDS because it does not appear to induce focused Treg responses, but rather can induce significant Th2 responses and even pathogenic Th1 and Th17 responses (reviewed in [30,31]). Of note, GAD-Alum (Diamyd Therapeutics) has been evaluated in several Phase I and II trials with anti-GAD65 antibody positive (Stage 2) or new-onset (Stage 3) subjects and showed trends toward preservation of residual insulin secretion, especially in subjects with late-onset autoimmune diabetes of adulthood (LADA) [27], but failed this trend in Phase III trials [32,33]. This clinical experience underscores a major problem in the preclinical development of ASIs in that GAD-Alum was never tested in animal models prior to clinical evaluation, and positive outcomes of GAD65 efficacy evaluations in NOD mouse efficacy studies were in a “prevention” setting with young (4- to 6-week-old) NOD mice but did not show reversal of the hyperglycemic Stage 2 condition [30]. Interestingly, in a prospective NOD study, the clinical GAD-Alum preparation did not prevent diabetes in the NOD mouse model [30]. Therefore, it is important to develop more regulatory-specific and potent TDSs such as soluble or particulate tolerance vehicles (e.g., nanoparticles, microspheres, and liposomes) containing different tolerogenic agents such as rapamycin, aryl-hydrocarbon receptor ligands, retinoic acid, vitamin D3, and cytokines such as interleukin (IL)-10 and transforming growth factor (TGF)-β [31,34]. Other TDSs are of a cellular nature in which tol-DCs or Tregs produced ex vivo are reintroduced in vivo [35,36], or are genetically modified gastrointestinal bacterial strains expressing autoantigen and tolerogenic cytokines [37]. Note that apoptotic tolerance vehicles are in this cellular class of TDSs.

While it is difficult to speculate which type of ASI will be most effective, apoptotic-based ASIs are appealing because they utilize a “natural” rather than synthetic tolerance system that avoids the risk of inducing pathogenic autoimmune responses due to the non-inflammatory tolerogenic nature of apoptotic cells (unlike synthetic particles that have a tendency to trigger inflammatory processes [38]). Indeed, there is currently a significant interest in apoptotic-based ASI development using different approaches. One such ASI is a soluble therapeutic comprised of recombinant autoantigen conjugated to a linker molecule that selectively binds erythrocytes (i.e., red blood cells, RBC) via the surface marker, glycophorin A, and upon systemic delivery has shown potent efficacy in preventing diabetes in NOD mice [5,39]. Once autoantigen-bound RBCs enter their natural apoptotic process (eryptosis for non-nucleated RBCs), tolerogenic APCs recognize and process them for interaction with T cells. Note that RBCs have an exceptionally high turnover rate of about 100 billion cells per day, thus potentially delivering high levels of autoantigen-bound apoptotic vesicles to tolerogenic APCs with each dose of the ASI. Another RBC-based apoptotic therapy using the transpeptidase, sortase, to covalently attach autoantigens to RBCs ex vivo prior to reinfusion also showed efficacy in preventing diabetes in NOD mice [6]. In addition, ex vivo chemically-induced apoptosis of mouse splenocytes or human peripheral blood mononuclear cells (PBMCs) [4] demonstrated efficacy in the autoimmune conditions of experimental autoimmune encephalomyelitis and T1D in mice and multiple sclerosis in human trials [40]. Others are using liposomes containing tolerogenic apoptosis mimicry substances such as phosphatidylserine to deliver autoantigen to tol-DCs from human T1D subjects [41].

In considering these apoptotic-based therapies, ADi-100 has a strong potential to prevent, treat, or even cure T1D with its highly potent TDS features of hyper-methylated DNA and the BAX-induced in-body apoptotic tolerance pathway. Other qualities of ADi-100 such as utilization of a non-cell therapeutic approach, low cost of production, favorable storage profile, and the ability to frequently dose over a long period of time to achieve tolerance, make it an attractive clinical development candidate. Given the variety of apoptotic TDSs in development, a proof-of-concept clinical study with one of these apoptotic ASIs will be of great value to the field.

## Figures and Tables

**Table 1:** mBG analysis of ADi-100-treated NOD female mice that showed very high hyperglycemia on the first day of treatment (day 0).

Mouse ID	mBG on Tx Day 0 (mg/dL)	Age (Days) on Day 0	Occurrences ≥ 180 mg/dL Prior to Day 0	Occurrences ≥ 180 mg/dL from Day 1–35	Age (days) at T1D ^a^	Days from Day 0 to T1D	Islet Insulin Staining ^c^	mBG at Day 35 (Study Termination)
	ADi-100 1:4 Tx group							
1	245	175	1	35	186	11	negative	≥600
2	230	91	0	35	93	2	n/a	≥600
3	255	111	0	35	116	5	negative	≥600
4	245	109	0	29	126	2	negative	≥600
5	301	279	3	35	279	0	negative	≥600
mean	255^b^	153		34 ^b^	160	4		
SEM	12	35		1	34	2		
6	217	181	2	15	n.d.		n/a	
7	197	270	0	15	n.d.		negative	184
8	192	231	6	16	n.d.		negative	199
9	180	222	1	11	n.d.		negative	183
10	213	146	2	4	n.d.		n/a	
mean	200	210		12				
SEM	7	21		2				
	ADi-100 1:2 Tx group							
1	284	191	1	27	209	18	negative	511
2	214	139	2	27	168	29	negative	351
3	211	243	2	20	n.d.		+	150
4	185	179	1	7	n.d.		+	133
5	286	192	0	6	n.d.		+	139
6	212	159	0	13	n.d.		+	174
7	202	197	0	3	n.d.		n/a	135
8	202	180	1	3	n.d.		+	130
9	196	188	0	0	n.d.		n/a	151
mean	213	191		7.4				
SEM	13	10		2.6				

Female NOD mice were monitored daily for mBG in which each mouse received the first ADi-100 dose (day 0) when mBG was ≥ 180 mg/dL on at least two occasions or when the first occurrence of mBG was ≥ 200 mg/dL. Mice received weekly ADi-100 injections thereafter for a total of five injections. Daily mBG monitoring continued and mice were diagnosed with diabetes when ≥ 300 mg/dL on 2 occasions at least 7 days apart (^a^ values denote age at the first of the 2 mBG measurements). Gray shaded cells are diabetic “non-responders” and non-shaded cells are non-diabetic “responders”. The study ended at day 35, which was when 100% incidence of diabetes occurred in the untreated group (see Figure 2 for diabetes incidence values). ^b^ *p* = 0.008 (two-tailed unpaired Wilcoxon test) for mean age comparison and *p* < 0.001 (Poisson regression) for mean mBG occurrences comparison to the respective means of non-diabetic responder mice 6–10 in the ADi-100 1:4 group. The untreated control group (*n* = 12) showed a mean ± SEM mBG and age on day 0 of 282 ± 29 mg/dL and 120 ± 9 days, respectively, and age at type I diabetes (T1D) diagnosis of 136 ± 12 days. n.d., not diabetic. ^c^ Animals were euthanized at the end of the experiment and pancreata harvested and stained for insulin (see examples of positive and negative insulin staining in Figure 4).
